# Chondrocalcinose articulaire révélatrice d'une hypercalcémie hypocalciurique familiale: à propos d'une observation

**DOI:** 10.11604/pamj.2015.20.58.5828

**Published:** 2015-01-22

**Authors:** Faten Frikha, Mouna Snoussi, Raida Ben Salah, Hanen Loukil, Zouhir Bahloul

**Affiliations:** 1 Service de Médecine Interne, CHU Hédi Chaker, 3029 Sfax, Tunisie

**Keywords:** Hypercalcémie, Hypercalcémie hypocalciurique familiale, Chondrocalcinose, Hypercalcemia, Family hypocalciuric hypercalcemia, Chondrocalcinosis

## Abstract

L'hypercalcémie hypocalciurique familiale (HHF) est une maladie bénigne à transmission autosomique dominante, caractérisée par une hypercalcémie persistante béhigne, une hypocalciurie, et des concentrations de parathormone (PTH) normales ou modérément élevées, sans complication secondaire à l'hypercalcémie. Nous rapportons l'observation d'un patient ayant présenté une chondrocalcinose articulaire révélatrice d'une HHF. A travers cette observation nous essayons de décrire les aspects épidémiologiques, les caractéristiques cliniques, et paracliniques de cette association.

## Introduction

L'hypercalcémie hypocalciurique familiale (HHF), également appelée syndrome de Marx, est une maladie bénigne à transmission autosomique dominante, caractérisée par une hypercalcémie persistante béhigne, une hypocalciurie, et des concentrations de parathormone (PTH) normales ou modérément élevées, sans complication secondaire à l'hypercalcémie. Elle peut s'associer à une chondrocalcinose articulaire (CCA), qui peut être d'ailleurs révélatrice de cette maladie. Peu de cas de cette association ont été rapportés dans la littérature. Nous rapportons l'observation d'un patient ayant présenté une CCA révélatrice d'une HHF. A travers cette observation nous essayons de décrire les aspects épidémiologiques, les caractéristiques cliniques, et paracliniques de cette association.

## Patient et observation

**Figure 1:**
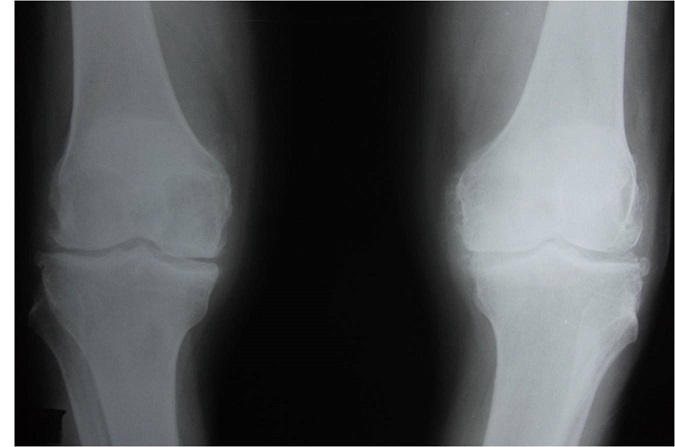
Radiographie des genoux: aspect de chondrocalcinose articulaire

Monsieur A.B, âgé de 69 ans, diabétique et hypertendu, était admis en orthopédie pour une arthrite aiguë de la cheville gauche avec fièvre. Il fut opéré en urgence pour suspicion d'arthrite septique mais l'examen bactériologique du liquide articulaire était négatif. Il était ensuite hospitalisé dans notre département devant l'installation d'une arthrite aiguë des 2 genoux. A l'examen clinique, il existait un épanchement des deux genoux. La température était normale et le reste de l'examen était sans particularités. La radiographie des genoux objectivait un liseré calcique au niveau de des interlignes articulaires (Figure 1) et la ponction ramenait un liquide riche en micro-cristaux de pyrophosphate de calcium dihydraté avec PNN non altérées. Le diagnostic d'une chondrocalcinose articulaire fut rapidement retenu. A la biologie il y'avait un syndrome inflammatoire avec une vitesse de sédimentation à 108 mm à la 1^ère^ heure, une CRP à 68,2 mg/l. L'hémogramme révélait une anémie à 9,6 g d'Hb normochrome normocytaire, des globules blancs à 5800 éléments/mm^3^ et des plaquettes à 353 000 éléments /mm^3^. Le bilan phosphocalcique trouvait une hypercalcémie confirmée à plusieurs reprises (3,02 - 2,89 - 2,97 mmol/l) avec phosphorémie et phosphatases alcalines normales avec une hypocalciurie à plusieurs reprises (0,69 mmol/24h). Devant cette hypercalcémie modérée asymptomatique, une enquête étiologique était entamée permettant d’éliminer une hyperparathyroïdie primaire (pas d'arguments biologiques en faveur, radiographie des mains et du crâne sans anomalies, échographie cervicale normale), un myélome multiple (absence de gammapathie monoclonale à l’électrophorèse des protéines sériques et myélogramme normal, absence d'insuffisance rénale), une sarcoïdose (radiographie thoracique normale, biopsie des glandes salivaires accessoires normale) et enfin des métastases osseuse (absence de douleurs osseuses, normalité des phosphatases alcalines, absence de signes de cancer). A l'interrogatoire, le patient ne prenait pas de médicament potentiellement responsable d'une hypercalcémie. Le diagnostic retenu était celui d'hypercalcémie hypocalciurique familiale (hypercalcémie bien toléré avec calciurie basse en l'absence d'autres étiologies d'hypercalcémie). Le patient était mis sous anti-inflammatoires non stéroïdiens et Colchicine avec infiltration locale de corticoïdes au niveau des genoux. L’évolution était favorable.

## Discussion

L’âge de notre patient au moment du diagnostic de la CCA était de 69 ans. Généralement à cet âge, on considère que la chondrocalcinose est idiopathique [1]. En effet, la fréquence des formes sporadiques augmente linéairement avec l’âge, et leur prévalence peut atteindre 20% au-delà de 80 ans. Les formes familiales ou associées à d'autres maladies métaboliques sont plus rares. Certaines maladies ont un lien de causalité bien défini avec la CCA, ce sont l'hyperparathyroïdie primitive, l'hémochromatose, l'hypomagnésémie et l'hypophosphatasie. D'autres affections ont été décrites en association avec la CCA, le plus souvent sur la base de données observationnelles, mais leur association est moins bien établie, c'est le cas de l'hypercalcémie hypocalciurique familiale (HHF) [1–3]. L'hypercalcémie est une anomalie fréquemment rencontrée en pratique clinique. Les principales maladies susceptibles de donner une hypercalcémie sont l'hyperparathyroïdie primitive et les cancers, qui représentent plus de 90% des cas. Les autres causes importantes d'hypercalcémie sont les traitements (principalement les diurétiques thiazidiques, le lithium et la vitamine D). Toutes ces étiologies ont pu être éliminées chez notre patient par une enquête exhaustive. Le profil biologique (hypercalcémie modérée ave hypocalciurie) a permis de retenir le diagnostic d'HHF. L'HHF est une maladie à transmission autosomique dominante, associée à des mutations du gène codant pour le récepteur sensible au calcium entraînant une perte de sa fonction. Il existe très peu de données sur l’épidémiologie de l'HHF et sa prévalence est estimée à 1 sur 78 000 dans une étude unique réalisée dans l'Est de l'Ecosse [4]. Pour confirmer le diagnostic d'HHF, la calcémie et la calciurie doivent être dosées chez les autres membres de la famille. La mutation du gène codant pour la maladie peut également être recherchée [5]. Cette recherche n'a pas pu être réalisée chez notre patient. L'association CCA-HHF a été rapportée à travers quelques observations [1, 6]. L'hypercalcémie chronique semble induire une CCA par une nucléation augmentée de pyrophosphate inorganique. Il est important de différentier l'HHF des autres causes d'hypercalcémie car c'est une maladie bénigne qui ne nécessite généralement pas de traitement.

## Conclusion

L'association HHF et CCA est rare mais possible. Une CCA secondaire à l'hypercalcémie chronique chez un sujet âgé peut être le signe clinique révélateur de cette maladie bénigne comme le montre notre observation. Cependant, une enquête étiologique de l'hypercalcémie à cet âge est indispensable afin de ne pas méconnaitre une pathologie maligne sous-jacente.
